# Osteoarthritis and hypertension: observational and Mendelian randomization analyses

**DOI:** 10.1186/s13075-024-03321-w

**Published:** 2024-04-17

**Authors:** Zhi-Jie Yang, Yuan Liu, Yan-Li Liu, Bin Qi, Xin Yuan, Wan-Xin Shi, Liu Miao

**Affiliations:** Departments of Cardiology, Liuzhou People’s Hospital, 8 Wenchang Road, Liuzhou, Guangxi, 545006 People’s Republic of China

**Keywords:** Osteoarthritis, Hypertension, Mendelian randomization, Observational research

## Abstract

**Background:**

The association between osteoarthritis (OA) and hypertension is a subject of ongoing debate in observational research, and the underlying causal relationship between them remains elusive.

**Methods:**

This study retrospectively included 24,871 participants in the National Health and Nutrition Examination Survey (NHANES) from 2013 to 2020. Weighted logistic regression was performed to investigate the connection between OA and hypertension. Additionally, Mendelian randomization (MR) analysis was conducted to explore the potential causal relationship between OA and hypertension.

**Results:**

In the NHANES data, after adjusting for multiple confounding factors, there was no significant relationship between OA and hypertension (OR 1.30, 95% CI, 0.97–1.73, *P* = 0.089). However, among males, OA appeared to be associated with a higher risk of hypertension (OR 2.25, 95% CI, 1.17–4.32, *P* = 0.019). Furthermore, MR results indicate no relationship between multiple OA phenotypes and hypertension: knee OA (IVW, OR 1.024, 95% CI: 0.931–1.126, *P* = 0.626), hip OA (IVW, OR 0.990, 95% CI: 0.941–1.042, *P* = 0.704), knee or hip OA (IVW, OR 1.005, 95% CI: 0.915–1.105, *P* = 0.911), and OA from UK Biobank (IVW, OR 0.796, 95% CI: 0.233–2.714, *P* = 0.715). Importantly, these findings remained consistent across different genders and in reverse MR.

**Conclusions:**

Our study found that OA patients had a higher risk of hypertension only among males in the observational study. However, MR analysis did not uncover any causal relationship between OA and hypertension.

**Supplementary Information:**

The online version contains supplementary material available at 10.1186/s13075-024-03321-w.

## Introduction

With the aging of the population, osteoarthritis (OA) has become a leading cause of disability and chronic pain in the elderly [1]. Its pathological characteristics include cartilage degeneration, bone remodeling, osteophyte formation, and synovial inflammation, which ultimately lead to pain, joint stiffness, swelling, and the eventual loss of normal joint function [2]. According to statistical data, approximately 80% of patients with knee or hip osteoarthritis experience varying degrees of activity limitations, and 25% unable to perform essential daily life activities [3].

Hypertension is a prominent global health concern, characterized by a continuous increase in its prevalence. In 2015, it was estimated that there were approximately 1.13 billion cases of hypertension worldwide. The prevalence of hypertension significantly rises with age, affecting over 60% of individuals aged over 60 years [4]. It has been extensively linked to cardiovascular diseases, including coronary artery disease (CAD), congestive heart failure (CHF), stroke, myocardial infarction (MF), atrial fibrillation (AF), and peripheral arterial disease (PAD), as well as kidney damage, Alzheimer’s disease, and other serious complications [5–7]. And efficient prevention and management of hypertension play a crucial role in reducing the global disease burden and promoting overall longevity [8]. Consequently, hypertension research has garnered substantial attention, with the aim of gaining a better understanding of its etiology, risk factors, and connections with other medical conditions. Ultimately, these efforts seek to develop more effective interventions and treatment strategies.

However, there is still controversy surrounding the potential link between OA and hypertension. Evidence suggests that approximately 55% of knee osteoarthritis patients aged 65 and older have hypertension, and Incident OA occurred more frequently with an increase in blood pressure level, even after adjusting for confounding factors such as body mass index (BMI) [9, 10].Nevertheless, some studies present differing viewpoints [11–14]. In order to gain a deeper understanding of the potential connection between OA and hypertension, this study aims to investigate the relationship between them using observational data. Furthermore, it seeks to assess the causal relationship between the two through bidirectional Mendelian randomization (MR) analysis.

## Methods

### Study design and study overview

**Fig. 1:**
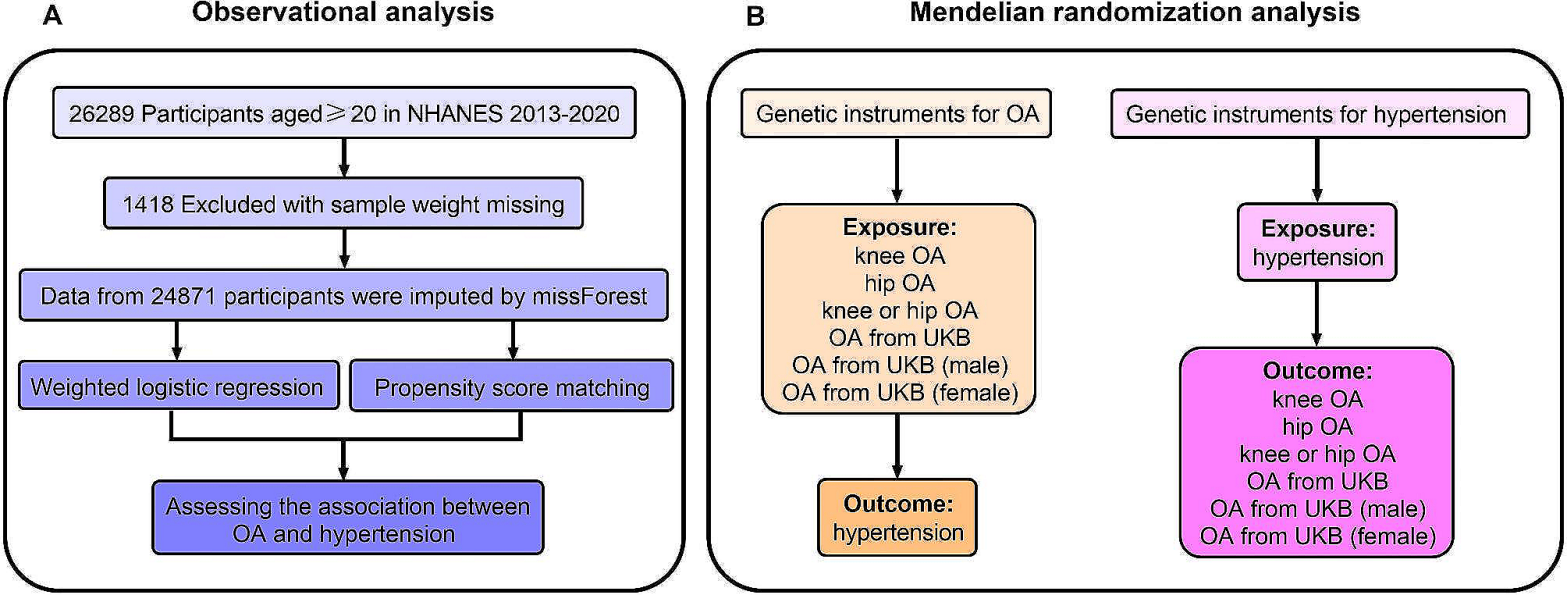
Overall Study Design Based on Observational Analysis and Mendelian Randomization. (**A**) The flowchart of the observational analysis. (**B**) The flowchart of the mendelian randomization analysis. OA, osteoarthritis; UKB, UK Biobank

This study was conducted in two phases. Initially, we characterized observational associations between osteoarthritis and hypertension using data derived from the National Health and Nutrition Examination Survey (NHANES). Subsequently, to investigate the potential causal relationship between osteoarthritis and hypertension, bidirectional two-sample Mendelian randomization (MR) analysis was performed using summary statistics from the genome-wide association study (GWAS). The forward MR analyses considered osteoarthritis as the exposure and hypertension as the outcome, while the reverse MR analyses hypertension as the exposure and osteoarthritis as the outcome. The flowchart is shown in Fig. 1.

### Observational analyses

#### Data sources and study population

NHANES constitutes an ongoing series of cross-sectional surveys aimed at evaluating the health and nutritional status of the civilians in the United States. This initiative is overseen by the National Center for Health Statistics under the purview of the Centers for Disease Control and Prevention (CDC). Additionally, it has received the ethical approval of the Ethics Review Board at the National Center for Health Statistics [15]. All procedures were performed following the guidelines of the Declaration of Helsinki.

In order to investigate the potential connection between osteoarthritis and hypertension, we conducted a retrospective analysis using data gathered from participants (≥ 20 years of age) across four 2-year NHANES survey cycles (2013–2020). The proportion of missing values for all variables was less than 15%, and missing data were imputed using the package “missforest” in R.

#### Diagnosis of osteoarthritis and hypertension

Osteoarthritis was denoted as M19.9 based on the International Classification of Diseases 10th Revision (ICD-10). Hypertension was defined as the average of three systolic blood pressure measurements ≥ 140 mmHg, the average of three diastolic blood pressure measurements ≥ 90 mmHg, current usage of medications for blood pressure control, or previously told had high blood pressure [16].

#### Assessment of covariates

The following covariates were included in this study: age, gender, NHANES cycles, race, educational level, family income, heavy drinker, smoker, BMI, uric acid, high-density lipoprotein cholesterol (HDL-C), total cholesterol (TC), triglyceride (TG), blood urea nitrogen (BUN), aspartate transaminase (AST), alanine aminotransferase (ALT), glycohemoglobin, estimate glomerular filtration rate (eGFR), cancer, diabetes mellitus (DM), osteoporosis, stroke, arteriosclerotic cardiovascular disease (ASCVD), heart attack, CHF, walk or bicycle, work activity, recreational activity, antiplatelet agents, statins, antihypertensive agents. Family income was divided into three levels according to the family poverty income ratio: low income (≤ 1.3), medium income (> 1.3 to 3.5), and high income (> 3.5) [17]. The definition of smoker, heavy drinker, ASCVD, and DM as described in previous literature [18]. Data on diagnosed history of cancer, osteoporosis, stroke, heart attack, and CHF were self-reported. Physical activity including walk or bicycle, work activity, and recreational activity was obtained in questionnaire. Specifically, work activity and recreational activity were classified into four categories based on NHANES guidelines: none, moderate, both, and vigorous. The use of antiplatelet agents, statins, and antihypertensive agents was derived from medication history.

#### Statistical analysis

According to NHANES analytic guidelines, complex sampling design and sampling weights were considered in this study [19]. T tests and Chi-square tests were applied for analyzing the association of hypertension with continuous variables and categorical variables, respectively. The multivariable logistic regression was employed to compute odds ratios (ORs) and 95% confidence intervals (95% CIs) to ascertain the effects of osteoarthritis on hypertension. In the logistic regression model, Model 1 was adjusted for none. Model 2 was adjusted for age, gender, heavy drinker, smoker, and education level. Model 3 was further adjusted for BMI, HDL-C, TC, BUN, ALT, glycohemoglobin, eGFR, family income, cancer, ASCVD, DM, osteoporosis, stroke, heart attack, CHF, uric acid, antiplatelet agents, and statins. Model 4 was based on model 3 and walk or bicycle. Confounding factors that were significantly associated with hypertension (*P* < 0.05) or a change in effect estimate of more than 10% were included in Model 4 [20]. The variance inflation factor (VIF) for all variables in the models was below 10, mitigating the impact of multicollinearity on the results [21]. Subgroup analyses were performed to evaluate the association between osteoarthritis and hypertension across various subgroups. And likelihood ratio tests were employed to identify potential interactions. Subsequently, propensity score matching (PSM) was conducted, the matching variables encompassed age, gender, heavy drinker, smoker, Race, education level, BMI, NHANES cycles, and walk or bicycle with caliper values set at 0.02. Final, logistic regression analysis was performed on the data after PSM. Model 1was adjusted for none. Model 2 was adjusted for age, gender, BMI, HDL-C, TG, BUN, ALT, AST, glycohemoglobin, eGFR, family income, uric acid, ASCVD, DM, stroke, heart attack, CHF, antiplatelet agents, recreational activity and statins. All analyses were performed using R 4.1.2 (http://www.r-project.org). Two-sided levels of significance were calculated, and the significance level was set as 0.05.

### Mendelian randomization

#### Selection of genetic instruments for MR analyses

To ensure the accuracy and robustness of causal inference, the selection of genetic instruments should satisfy the three key assumptions of MR. First, Single Nucleotide Polymorphisms (SNPs) were chosen based on a genome-wide significance threshold and F-statistics > 10 [22]. Second, independent SNPs were kept based on linkage disequilibrium (LD) as measured by r^2^ < 0.001 and clumping distance = 10,000 kb. Final, SNPs associating with the outcome and confounding factors (*p* < 5 × 10^− 8^) were excluded, and palindromic SNPs with intermediate allele frequencies were deleted when harmonizing exposure and outcome data [23, 24].

#### Data sources and genetic instruments selection for osteoarthritis

The OA data were sourced from the UK Biobank (UKB) [25], which serves as a crucial biobank resource (http://www.nealelab.is/uk-biobank/) and from the GWAS data analyzed by Tachmazidou et al. [26]. In the dataset obtained from Tachmazidou, three distinct phenotypes were employed in our study: knee osteoarthritis, hip osteoarthritis, and knee or hip osteoarthritis, as detailed in Table S1. When osteoarthritis was considered as the exposure variable, the significance threshold was set at *p* < 5 × 10^− 7^. SNPs associated with hypertension or with confounding factors such as body weight, BMI, and obesity were systematically excluded through the use of PhenoScanner [27].

#### Data sources and genetic instruments selection for hypertension

The sources of GWAS data for hypertension encompassed the ninth release of the FinnGen Study [28]. In the FinnGen Study, the hypertension dataset consisted of 111,581 cases and 265,626 controls. When hypertension was considered as the exposure, we employed a more stringent significance threshold (*p* < 1 × 10^− 8^) due to the numerous SNPs associated with hypertension. Furthermore, SNPs associated with osteoarthritis or with confounding factors (bone density, body weight, BMI, obesity, and diabetes) were excluded using PhenoScanner.

#### Statistical analysis for MR

Heterogeneity for both inverse-variance weighted (IVW) and MR-Egger methods was evaluated through Cochran’s Q statistics. In light of observed potential heterogeneity, this investigation adopted the random-effects IVW model as the primary analytical approach to explore the causal association between osteoarthritis and hypertension [29]. And other MR methods, such as MR Egger, weighted median, simple mode, and weighted mode, were also employed as reference points. Furthermore, to bolster the assessment of the causal relationship and alleviate the potential influence of horizontal pleiotropy on the outcomes, MR-Egger methods were employed [30, 31]. Moreover, if MR-Egger methods detect horizontal pleiotropy in the genetic instruments, additional steps will be taken to address this issue. These include conducting an outlier test using MR-PRESSO, and subsequently, any outlying SNPs identified in the MR-PRESSO outlier test will be excluded [32]. This process aims to further mitigate the impact of pleiotropic SNPs. All MR analyses were performed using the package “TwosampleMR” [33](version 0.5.6) in R.

## Result

### Population characteristics of NHANES

This study comprised a total of 24,871 participants after excluding 1,418 individuals with missing sample weight data. The weighted mean age was 48.05 years (95% CI, 51.17–52.63 years), and females accounted for 51.90% (95% CI, 51.17-52.63%). The main characteristics are summarized in Table S2. Among all participants, 360 individuals were diagnosed with OA, representing 1.64% of the total participants, while 10,546 individuals had hypertension, constituting 37.78% of the participants. In comparison to those without OA, individuals with OA exhibited higher age, a greater proportion of females, and an increased BMI. Additionally, they demonstrated a heightened prevalence of conditions including cancer, ASCVD, DM, osteoporosis, stroke, CHF, and hypertension. Furthermore, they reported lower levels of physical activity, as indicated in Table 1.

**Table 1:** Baseline Characteristics across OA Strata of Participants in NHANES 2013–2020

Variables	Non-OA	OA	P value
Age, years	47.79 (47.29 ,48.29)	63.08 (61.64 ,64.53)	< 0.001
Gender, %			< 0.001
Male	48.48 (47.74 ,49.22)	25.08 (19.36 ,31.82)	
Female	51.52 (50.78 ,52.26)	74.92 (68.18 ,80.64)	
NHANES cycles, %			0.919
2013–2014	24.28 (22.24 ,26.45)	25.55 (18.61 ,34.00)	
2015–2016	24.91 (22.76 ,27.18)	22.82 (16.53 ,30.64)	
2017–2018	25.32 (23.71 ,27.01)	25.09 (18.96 ,32.41)	
2019–2020	25.49 (23.66 ,27.40)	26.54 (20.46 ,33.65)	
Race, %			< 0.001
Mexican American	8.86 (7.30 ,10.71)	3.59 (1.97 ,6.46)	
Non-Hispanic White	63.39 (60.24 ,66.42)	76.27 (70.28 ,81.37)	
Non-Hispanic Black	11.50 (9.89 ,13.35)	6.80 (4.77 ,9.61)	
Other Hispanic	6.72 (5.80 ,7.77)	4.01 (2.59 ,6.15)	
Other Race	9.54 (8.47 ,10.72)	9.34 (6.58 ,13.08)	
Educational level, %			0.658
< High school	13.04 (11.86 ,14.31)	13.87 (10.47 ,18.16)	
High school	24.15 (22.87 ,25.48)	26.34 (19.53 ,34.50)	
Some college or more	62.81 (60.79 ,64.80)	59.79 (52.55 ,66.62)	
Family income, %			0.593
Low	22.00 (20.47 ,23.61)	22.92 (17.56 ,29.34)	
Medium	35.07 (33.64 ,36.53)	31.56 (25.30 ,38.57)	
High	42.93 (40.65 ,45.24)	45.52 (38.25 ,52.98)	
Heavy drinker, %			0.573
No	71.33 (69.93 ,72.69)	72.10 (66.09 ,77.41)	
Yes	13.03 (12.25 ,13.85)	14.61 (10.18 ,20.52)	
Missing	15.65 (14.51 ,16.85)	13.29 (8.94 ,19.32)	
Smoker, %			0.048
No	81.81 (80.70 ,82.87)	86.68 (81.94 ,90.33)	
Yes	18.19 (17.13 ,19.30)	13.32 (9.67 ,18.06)	
BMI, kg/m2	29.52 (29.31 ,29.74)	31.71 (30.61 ,32.81)	< 0.001
Uric acid, umol/l	319.96 (318.43 ,321.48)	310.29 (297.93 ,322.66)	0.135
HDL-C, mmol/l	1.40 (1.38 ,1.41)	1.50 (1.44 ,1.56)	0.002
TC, mmol/l	4.90 (4.87 ,4.93)	4.99 (4.84 ,5.15)	0.238
TG, mmol/l	1.66 (1.62 ,1.69)	1.75 (1.61 ,1.89)	0.225
BUN, mmol/l	5.15 (5.09 ,5.21)	6.15 (5.88 ,6.41)	< 0.001
ALT, u/l	23.95 (23.61 ,24.29)	24.13 (21.80 ,26.45)	0.884
AST, u/l	23.68 (23.43 ,23.94)	22.52 (21.56 ,23.48)	0.021
glycohemoglobin, %	5.66 (5.64 ,5.68)	5.74 (5.64 ,5.85)	0.122
eGFR, mL/min per 1.73m^2^	94.61 (93.90 ,95.32)	80.69 (78.35 ,83.03)	< 0.001
Cancer, %			0.027
No	88.96 (88.36 ,89.53)	83.56 (76.95 ,88.55)	
Yes	11.04 (10.47 ,11.64)	16.44 (11.45 ,23.05)	
ASCVD, %			< 0.001
No	91.78 (91.15 ,92.36)	77.29 (71.05 ,82.51)	
Yes	8.22 (7.64 ,8.85)	22.71 (17.49 ,28.95)	
DM, %			< 0.001
No	86.08 (85.41 ,86.73)	76.79 (69.53 ,82.74)	
Yes	13.92 (13.27 ,14.59)	23.21 (17.26 ,30.47)	
Osteoporosis, %			< 0.001
No	29.90 (28.16 ,31.69)	53.28 (46.23 ,60.20)	
Yes	2.38 (2.10 ,2.70)	5.24 (2.83 ,9.48)	
Missing	67.72 (65.75 ,69.63)	41.49 (34.29 ,49.07)	
Stroke, %			< 0.001
No	97.00 (96.72 ,97.25)	87.38 (83.16 ,90.66)	
Yes	3.00 (2.75 ,3.28)	12.62 (9.34 ,16.84)	
Heart attack, %			0.260
No	96.49 (96.06 ,96.87)	95.34 (92.49 ,97.14)	
Yes	3.51 (3.13 ,3.94)	4.66 (2.86 ,7.51)	
CHF, %			< 0.001
No	97.58 (97.32 ,97.81)	93.46 (89.65 ,95.94)	
Yes	2.42 (2.19 ,2.68)	6.54 (4.06 ,10.35)	
Walk or bicycle, %			< 0.001
No	78.85 (77.66 ,79.99)	89.59 (84.27 ,93.25)	
Yes	21.15 (20.01 ,22.34)	10.41 (6.75 ,15.73)	
Work activity, %			0.026
No	51.40 (50.05 ,52.75)	63.68 (53.76 ,72.55)	
Moderate	24.40 (23.35 ,25.48)	19.30 (13.71 ,26.47)	
Both	20.19 (19.13 ,21.29)	13.55 (8.62 ,20.67)	
Vigorous	4.02 (3.56 ,4.53)	3.47 (1.70 ,6.95)	
Recreational activity, %			0.002
No	45.73 (44.05 ,47.41)	58.20 (49.49 ,66.43)	
Moderate	26.52 (25.45 ,27.61)	28.16 (20.83 ,36.86)	
Both	19.78 (18.57 ,21.05)	11.53 (6.61 ,19.35)	
Vigorous	7.97 (7.38 ,8.62)	2.12 (0.75 ,5.82)	
Antiplatelet agents, %			< 0.001
No	97.60 (97.29 ,97.87)	93.90 (90.36 ,96.19)	
Yes	2.40 (2.13 ,2.71)	6.10 (3.81 ,9.64)	
Statins, %			< 0.001
No	82.16 (81.17 ,83.10)	51.27 (43.79 ,58.70)	
Yes	17.84 (16.90 ,18.83)	48.73 (41.30 ,56.21)	
Antihypertensive agents, %			< 0.001
No	95.25 (94.78 ,95.67)	88.00 (82.31 ,92.04)	
Yes	4.75 (4.33 ,5.22)	12.00 (7.96 ,17.69)	
Hypertension, %			< 0.001
No	62.70 (61.35 ,64.04)	33.02 (27.60 ,38.93)	
Yes	37.30 (35.96 ,38.65)	66.98 (61.07 ,72.40)	

Variables are presented as weighted percentage (%) or mean (95% confidence interval)

BMI, body mass index; HDL-C, high-density lipoprotein cholesterol; TC, total cholesterol; TG, triglyceride; BUN, blood urea nitrogen; ALT, alanine aminotransferase; AST, aspartate transaminase; eGFR, estimated glomerular filtration rate; ASCVD, arteriosclerotic cardiovascular disease; DM, diabetes mellitus; CHF, congestive heart failure; OA, osteoarthritis

### Association between OA and hypertension in NHANES

The results from weighted logistic regression analysis indicated that, in the unadjusted model, individuals with osteoarthritis exhibited a higher hypertension risk compared to those without (OR 3.41, 95% CI, 2.64–4.40, *P* < 0.001). However, upon controlling for confounding factors in Model 3, this relationship ceased to be statistically significant (OR 1.30, 95% CI, 0.98–1.74, *P* = 0.080). Further adjustment for physical activity did not change the observed association between OA and hypertension (OR 1.30, 95% CI, 0.97–1.73, *P* = 0.089). The specific results are presented in Table 2.

**Table 2:** The Association of OA and Hypertension in NHANES 2013–2020

Model	OR (95% CI)	P value
Model 1	3.41 (2.64, 4.40)	< 0.001
Model 2	1.51 (1.15, 1.98)	0.004
Model 3	1.30 (0.98, 1.74)	0.080
Model 4	1.30 (0.97, 1.73)	0.089

OR, odds ratio; CI, confidence interval

Model 1: adjusted for none

Model 2: adjusted for age, gender, heavy drinker, smoker, and education level

Model 3: further adjusted (from Model 2) for body mass index, high-density lipoprotein cholesterol, total cholesterol, blood urea nitrogen, alanine aminotransferase, glycohemoglobin, estimated glomerular filtration rate, family income, cancer, arteriosclerotic cardiovascular disease, diabetes mellitus, osteoporosis, stroke, heart attack, congestive heart failure, uric acid, antiplatelet agents, and statins

Model 4: further adjusted (from Model 3) for walk or bicycle

**Fig. 2:**
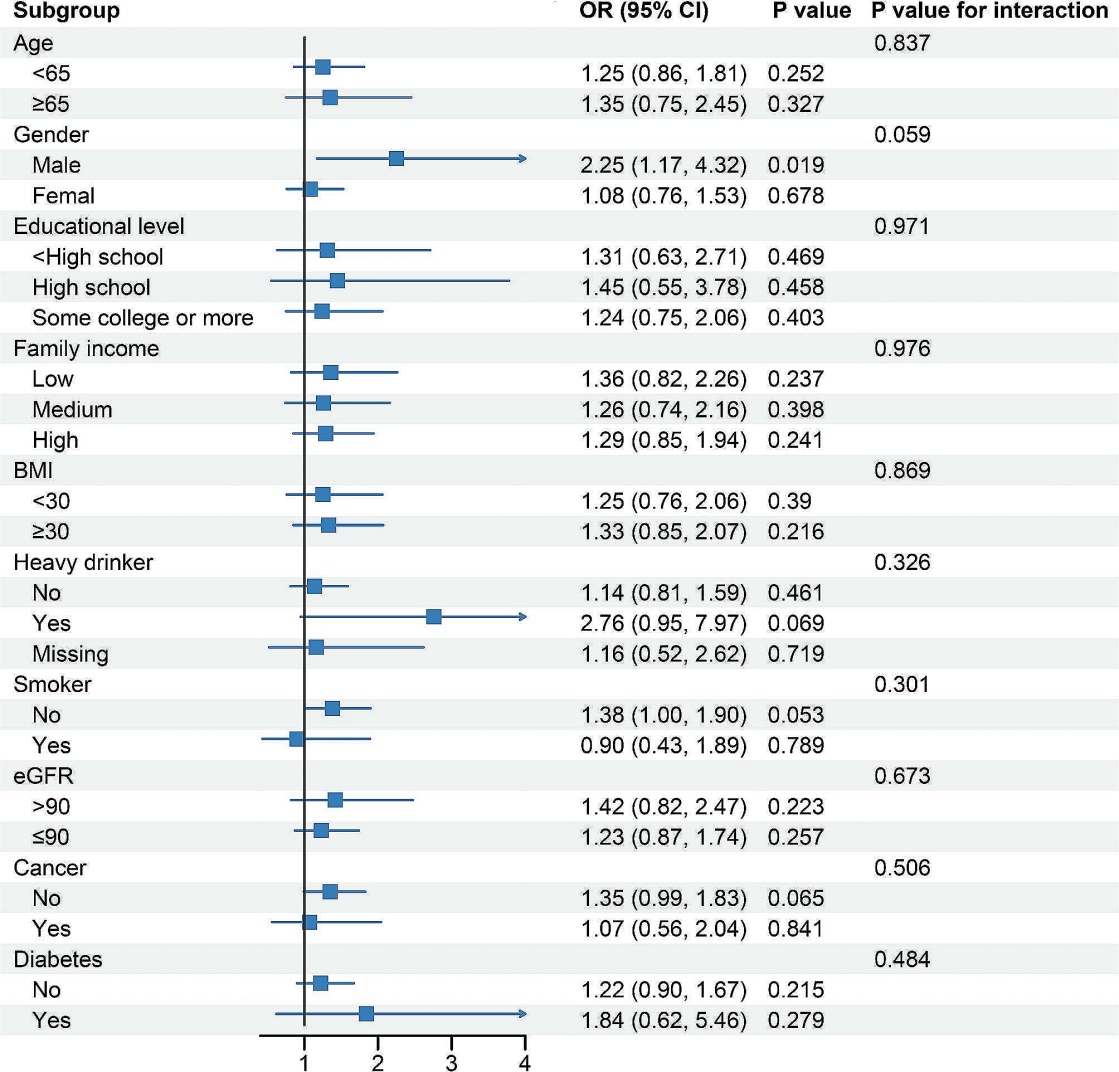
The Relationship between OA and Hypertension in Different Subgroups of NHANES 2013–2020. Each stratification was adjusted for variables in Model 4 except the stratification factor itself

In subgroup analyses, all subgroups adjusted for variables in Model 4 except for the stratification variable. The results of the subgroup analyses are depicted in Fig. 2. In the majority of subgroups, there was no significant association between OA and hypertension. It is noteworthy that among males, individuals with OA appeared to have a higher risk of hypertension (OR 2.25, 95% CI, 1.17–4.32, *P* = 0.019). However, interaction analysis indicated that there was no significant difference in the relationship between OA and hypertension across different genders.

To further investigate the association between OA and hypertension, PSM was conducted, followed by univariate and multivariate logistic regression analyses on the matched data. The results showed that even after PSM, a total of 13,192 participants remained, including 6,596 with hypertension and 219 with OA (Table S3). Whether through univariate or multivariate logistic regression, the results consistently failed to reveal a significant association between OA and hypertension (Table S4).

### Causal effects of OA on hypertension by MR

**Fig. 3:**
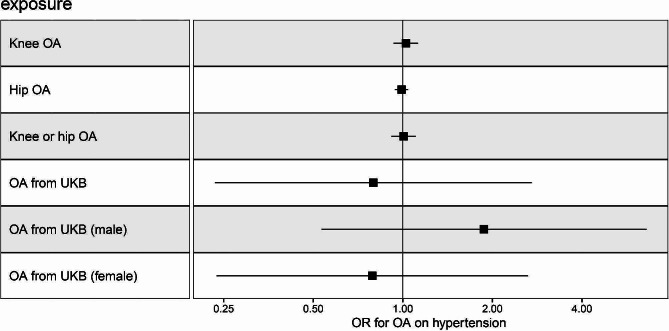
The Causal Relationship of OA on Hypertension. OR and 95% confidence interval were calculated using the inverse variance weighted method. OR, odds ratio; OA, osteoarthritis; UKB, UK Biobank

In our study, for each instrument, all F-statistics were > 10, indicating a reduced susceptibility to weak instrument bias and a higher confidence in the causal effect estimate. MR-Egger regression analysis did not reveal significant horizontal pleiotropy between OA and hypertension (*P* > 0.05). However, due to the presence of heterogeneity, using IVW as our primary assessment method is more appropriate. The horizontal pleiotropy and heterogeneity results for instrumental variables are shown in Tables S5 and S6. The results presented in Fig. 3 and Table S7 suggest the absence of a causal relationship between multiple OA datasets and hypertension: knee OA (IVW, OR 1.024, 95% CI: 0.931–1.126, *P* = 0.626), hip OA (IVW, OR 0.990, 95% CI: 0.941–1.042, *P* = 0.704), knee or hip OA (IVW, OR 1.005, 95% CI: 0.915–1.105, *P* = 0.911), and OA from UKB (IVW, OR 0.796, 95% CI: 0.233–2.714, *P* = 0.715). Moreover, alternative MR methods yielded similar findings, with none indicating any causal effects of OA on hypertension (both *P* > 0.05). Furthermore, in light of observations among male participants from NHANES suggesting that individuals with OA may exhibit an increased hypertension risk, we conducted an in-depth exploration of the causal effects of OA on hypertension within different gender subgroups using MR. The findings consistently indicated the absence of a causal relationship between the two in distinct gender groups: males (IVW, OR 1.874, 95% CI: 0.533–6.596, *P* = 0.328), and females (IVW, OR 0.789, 95% CI: 0.236–2.636, *P* = 0.701).

### Causal effects of hypertension on OA by MR

**Fig. 4:**
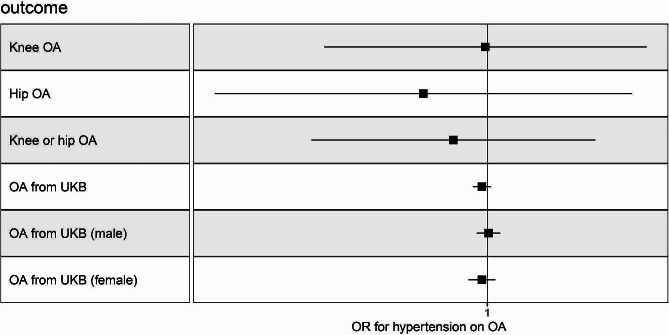
The Causal Relationship of Hypertension on OA. OR and 95% confidence interval were calculated using the inverse variance weighted method. OR, odds ratio; OA, osteoarthritis; UKB, UK Biobank

To explore the potential reverse causal relationship between OA and hypertension, we conducted a reverse MR analysis, using hypertension as the exposure variable and OA as the outcome variable. Similarly, in the reverse MR analysis, no evidence of horizontal pleiotropy was detected. Moreover, we did not observe any causal association between hypertension and various OA outcomes: knee OA (IVW, OR 0.999, 95% CI: 0.949–1.052, *P* = 0.981), hip OA (IVW, OR 0.980, 95% CI: 0.917–1.047, *P* = 0.548), knee or hip OA (IVW, OR 0.989, 95% CI: 0.946–1.035, *P* = 0.639), and OA from the UK Biobank (IVW, OR 0.998, 95% CI: 0.995–1.001, *P* = 0.251). Detailed results can be found in Fig. 4 and Table S8.

## Discussion

To the best of our knowledge, this study represents the first comprehensive investigation into the relationship between OA and hypertension risk, employing a combination of extensive observational study data and MR analysis based on large-scale genetic dataset. The outcomes of this research provide novel insights into the exploration of the connection between OA and hypertension. In the retrospective segment of the study, we included a substantial sample from the NHANES, comprising a total of 24,871 participants. Following meticulous weighting, this sample effectively represents the U.S. population. The findings initially suggest that, in unadjusted models, OA patients demonstrate a higher hypertension risk. However, upon further adjustment for confounding factors, this association loses its statistical significance. Even in subgroup analyses, with the exception of males, no substantial correlation between OA and hypertension was observed in other subgroups. These results are in alignment with prior literature [11–14].

However, numerous studies have also suggested that OA serves as a risk factor for hypertension. In a prospective cohort study, it was found that patients with knee osteoarthritis experienced a 13% increased risk of developing hypertension during an 8-year follow-up period. This association remained robust even after employing propensity score matching [34]. Furthermore, the findings of a meta-analysis underscore a significant connection between hypertension and knee joints, as opposed to non-weight-bearing joints [35]. These studies are theoretically grounded in the notion that OA patients often contend with chronic pain and restricted mobility, which can potentially lead to weight gain, reduced physical activity, and metabolic disruptions [36, 37], all of which are recognized risk factors for hypertension. Moreover, Research suggest that OA is typically characterized by a certain degree of chronic low-grade inflammation, with various soluble inflammatory mediators such as Interleukin-6 (IL-6), Tumour necrosis factor alpha (TNF-α), and C-reactive protein (CRP) elevated in osteoarthritic tissues [38]. Notably, inflammation plays a pivotal role in hypertension. It has been reported that IL-6 inhibition attenuates hypertension and blunts the infiltration or proliferation of macrophages and mononuclear cells into the kidneys, thereby reducing hypertension-related renal damage in Dahl salt-sensitive rats [39]. And inflammation is believed to be a contributing factor to the elevated risk of cardiovascular disease-related mortality in individuals with OA. Inflammatory processes may expedite the development of atherosclerosis, leading to hypertension by causing arterial stiffening and proliferation due to arterial wall degeneration [40]. However, it’s worth noting that current anti-inflammatory therapies have not proven to be highly effective, whether in treating osteoarthritis or hypertension [38, 41].

In our retrospective study, we observed a heightened risk of hypertension among males with osteoarthritis, while no such association was observed in females. Androgen signaling may play a crucial role in this gender disparity, as previous studies have shown that increased androgen receptor (AR) activity and testosterone levels may influence hypertension by altering the renin-angiotensin-aldosterone system [42]. And lower CAG repeat lengths in the AR coding region are associated with central obesity and hypertension in males rather than females [43]. Additionally, study have suggested an association between androgen receptor polymorphism and osteoarthritis [44], and recent Mendelian randomization studies have further indicated a causal relationship between androgen and the occurrence of osteoarthritis [45]. Therefore, androgen signaling may partially explain the association between osteoarthritis and hypertension. Nonetheless, observational studies cannot conclusively determine whether this association is merely correlational or causal.

Owing to potential confounding factors and bidirectionality inherent in observational data, this may account for the disparities between our study and others. In order to bolster the control over confounders and thereby bolster more robust causal inferences, we conducted MR analysis to explore the causal relationships between different subtypes of osteoarthritis and hypertension within distinct gender groups. However, we found no association between the genetic liability to osteoarthritis and hypertension. This consistency was observed across different genders and in reverse analyses.

There is currently limited fundamental research on the connection between OA and hypertension. It has been reported that knee OA patients with comorbid hypertension and diabetes exhibit significant bone loss in the subchondral plate, with the medial part of the tibial plateau demonstrating lower bone mineral density (BMD) and higher porosity [46]. Additionally, in OA rats with coexisting hypertension, there is a higher level of synovial inflammation, which may potentially facilitate the progression of OA [47]. Nevertheless, these findings are still insufficient to explain whether OA leads to the development of hypertension or if hypertension contributes to the onset of OA. Overall, based on our research findings, there may not be a causal relationship between OA and hypertension.

This study has several strengths. We employed a combined approach of observational research and MR analysis to investigate the relationship between OA and hypertension. This comprehensive research design allows for a more holistic understanding of the connection between OA and hypertension, supported by multiple lines of evidence. Nonetheless, the study does come with certain limitations. Firstly, despite using a large-scale NHANES sample, the prevalence of OA is relatively low, which might affect our ability to detect causal relationships. Secondly, even though we controlled for various confounding factors in the study, there may still be unaccounted confounders that could introduce potential interference when explaining the relationship between OA and hypertension. And in the retrospective study, we did not further analyze the association between different subtypes of OA and hypertension. Moreover, although there was no pleiotropy in the MR analyses, some exhibited heterogeneity, which could potentially impact our results.

## Conclusions

In the MR analysis, no causal relationship was found between OA and hypertension, even though the observational study revealed a higher risk of hypertension in the male subgroup of OA patients. However, it’s important to note that the observational findings might be subject to bias due to uncontrolled confounders. Therefore, it is essential to conduct more well-designed prospective studies to minimize observational study biases and redundancy. Furthermore, utilizing larger GWAS datasets for MR analysis is recommended.

### Electronic supplementary material

Below is the link to the electronic supplementary material.

Supplementary Material 1

## Data Availability

No datasets were generated or analysed during the current study.

## Abbreviations

AF: Atrial fibrillation
ASCVD: Arteriosclerotic cardiovascular disease
AST: Aspartate transaminase
ALT: Alanine aminotransferase
BUN: Blood urea nitrogen
BMI: Body mass index
BMD: Bone mineral density
CAD: Coronary artery disease
CHF: Congestive heart failure
CDC: Centers for Disease Control and Prevention
CRP: C-reactive protein
CI: Confidence interval
DM: Diabetes mellitus
eGFR: Estimate glomerular filtration rate
GWAS: Genome-wide association study
HDL-C: High-density lipoprotein cholesterol
IVW: Inverse-variance weighted
ICD-10: International Classification of Diseases 10th Revision
LD: Linkage disequilibrium
IL-6: Interleukin-6
MR: Mendelian randomization
MF: Myocardial infarction
NHANES: National Health and Nutrition Examination Survey
OA: Osteoarthritis
OR: Odds ratio
PAD: Peripheral arterial disease
SNPs: Single Nucleotide Polymorphisms
TC: Total cholesterol
TG: Triglyceride
TNF-α: Tumour necrosis factor alpha
UKB: UK Biobank
