# Supplementary material for: Osteoarthritis and hypertension: observational and Mendelian randomization analyses
Source: Arthritis Res Ther. 2024 Apr 17;26:88. doi: 10.1186/s13075-024-03321-w (PMC11022320; doi:10.1186/s13075-024-03321-w)
Supplement: Supplementary file 1 — Supplementary Material 1 [file 13075_2024_3321_MOESM1_ESM.docx]

**Table S1**. Characteristics of Summary Datasets for Osteoarthritis and Hypertension

| **Phenotypes** | **Sample size** | **Control** | **Case** | **Prevalence (%)** |
| --- | --- | --- | --- | --- |
| Knee OA | 403,124 | 378,169 | 24,955 | 6.19 |
| Hip OA | 393,873 | 378,169 | 15,704 | 3.99 |
| Knee or hip OA | 417,596 | 378,169 | 39,427 | 9.44 |
| OA from UKB | 361,141 | 331,095 | 30,046 | 8.32 |
| OA from UKB (male) | 166,988 | 156,339 | 10,649 | 6.38 |
| OA from UKB (female) | 194,153 | 174,756 | 19,397 | 9.99 |
| Hypertension | 377,207 | 265,626 | 111,581 | 29.58 |

OA, osteoarthritis; UKB, UK Biobank.

**Table S2**. Baseline Characteristics of Participants in NHANES 2013-2020.

| **Variables** | **Participants** | **Percentage / Mean(95%CI)** |
| --- | --- | --- |
| Age, years | 24871 | 48.05 (47.55 ,48.54) |
| Gender, % |  |  |
| Male | 11969 | 48.10 (47.37 ,48.83) |
| Female | 12902 | 51.90 (51.17 ,52.63) |
| NHANES cycles, % |  |  |
| 2013-2014 | 5588 | 24.30 (22.24 ,26.49) |
| 2015-2016 | 5474 | 24.87 (22.73 ,27.14) |
| 2017-2018 | 5265 | 25.32 (23.69 ,27.03) |
| 2019-2020 | 8544 | 25.50 (23.66 ,27.44) |
| Race, % |  |  |
| Mexican American | 3383 | 8.77 (7.22 ,10.61) |
| Non-Hispanic White | 8927 | 63.60 (60.45 ,66.63) |
| Non-Hispanic Black | 5839 | 11.43 (9.81 ,13.27) |
| Other Hispanic | 2601 | 6.67 (5.76 ,7.72) |
| Other Race | 4121 | 9.54 (8.48 ,10.70) |
| Educational level, % |  |  |
| <High school | 5187 | 13.05 (11.87 ,14.32) |
| High school | 5757 | 24.19 (22.90 ,25.52) |
| Some college or more | 13927 | 62.76 (60.74 ,64.74) |
| Family income, % |  |  |
| Low | 8006 | 22.02 (20.51 ,23.60) |
| Medium | 9446 | 35.01 (33.59 ,36.46) |
| High | 7419 | 42.97 (40.71 ,45.26) |
| Heavy drinker, % |  |  |
| No | 16741 | 71.34 (69.94 ,72.70) |
| Yes | 3255 | 13.05 (12.28 ,13.87) |
| Missing | 4875 | 15.61 (14.46 ,16.83) |
| Smoker, % |  |  |
| No | 20188 | 81.89 (80.81 ,82.93) |
| Yes | 4683 | 18.11 (17.07 ,19.19) |
| BMI, kg/m2 | 24871 | 29.56 (29.34 ,29.77) |
| Uric acid, umol/l | 24871 | 319.80 (318.29 ,321.31) |
| HDL-C, mmol/l | 24871 | 1.40 (1.39 ,1.41) |
| TC, mmol/l | 24871 | 4.90 (4.87 ,4.94) |
| TG, mmol/l | 24871 | 1.66 (1.62 ,1.69) |
| BUN, mmol/l | 24871 | 5.17 (5.11 ,5.23) |
| ALT, u/l | 24871 | 23.95 (23.62 ,24.28) |
| AST, u/l | 24871 | 23.66 (23.41 ,23.92) |
| glycohemoglobin, % | 24871 | 5.66 (5.64 ,5.68) |
| eGFR, mL/min per  1.73m^2^ | 24871 | 94.38 (93.67 ,95.09) |
| Cancer, % |  |  |
| No | 22357 | 88.87 (88.27 ,89.44) |
| Yes | 2514 | 11.13 (10.56 ,11.73) |
| ASCVD, % |  |  |
| No | 22271 | 91.54 (90.93 ,92.11) |
| Yes | 2600 | 8.46 (7.89 ,9.07) |
| DM, % |  |  |
| No | 20205 | 85.93 (85.22 ,86.61) |
| Yes | 4666 | 14.07 (13.39 ,14.78) |
| Osteoporosis, % |  |  |
| No | 8296 | 30.28 (28.56 ,32.06) |
| Yes | 662 | 2.43 (2.14 ,2.75) |
| Missing | 15913 | 67.29 (65.33 ,69.20) |
| Stroke, % |  |  |
| No | 23779 | 96.84 (96.56 ,97.09) |
| Yes | 1092 | 3.16 (2.91 ,3.44) |
| Heart attack, % |  |  |
| No | 23754 | 96.47 (96.05 ,96.84) |
| Yes | 1117 | 3.53 (3.16 ,3.95) |
| CHF, % |  |  |
| No | 23982 | 97.51 (97.25 ,97.75) |
| Yes | 889 | 2.49 (2.25 ,2.75) |
| Walk or bicycle, % |  |  |
| No | 19102 | 79.03 (77.84 ,80.16) |
| Yes | 5769 | 20.97 (19.84 ,22.16) |
| Work activity, % |  |  |
| No | 14033 | 51.60 (50.27 ,52.93) |
| Moderate | 5299 | 24.31 (23.29 ,25.37) |
| Both | 4476 | 20.08 (19.02 ,21.17) |
| Vigorous | 1063 | 4.01 (3.56 ,4.50) |
| Recreational activity, % |  |  |
| No | 13018 | 45.93 (44.27 ,47.60) |
| Moderate | 6051 | 26.54 (25.48 ,27.64) |
| Both | 4030 | 19.65 (18.42 ,20.93) |
| Vigorous | 1772 | 7.88 (7.29 ,8.50) |
| Antiplatelet agents, % |  |  |
| No | 24077 | 97.54 (97.25 ,97.79) |
| Yes | 794 | 2.46 (2.21 ,2.75) |
| Statins, % |  |  |
| No | 19687 | 81.65 (80.64 ,82.63) |
| Yes | 5184 | 18.35 (17.37 ,19.36) |
| Antihypertensive agents, % |  |  |
| No | 23431 | 95.13 (94.67 ,95.54) |
| Yes | 1440 | 4.87 (4.46 ,5.33) |
| Hypertension, % |  |  |
| No | 14325 | 62.22 (60.87 ,63.54) |
| Yes | 10546 | 37.78 (36.46 ,39.13) |
| OA |  |  |
| No | 24511 | 98.36 (98.10 ,98.58) |
| Yes | 360 | 1.64 (1.42 ,1.90) |

Variables are presented as weighted percentage (%) or mean (95% CI).

BMI, body mass index; HDL-C, high-density lipoprotein cholesterol; TC, total cholesterol; TG, triglyceride; BUN, blood urea nitrogen; ALT, alanine aminotransferase; AST, aspartate transaminase; eGFR, estimated glomerular filtration rate; ASCVD, arteriosclerotic cardiovascular disease;DM, diabetes mellitus; CHF, congestive heart failure; OA, osteoarthritis.

**Table S3.** Characteristics of Participants in NHANES 2013-2020 after Propensity Score Matching.

| **Variables** | **Participants** | **Percentage / Mean(95%CI)** |
| --- | --- | --- |
| Age, years | 13192 | 52.96 (52.46 ,53.46) |
| Gender, % |  |  |
| Male | 6627 | 50.10 (49.04 ,51.16) |
| Female | 6565 | 49.90 (48.84 ,50.96) |
| NHANES cycles, % |  |  |
| 2013-2014 | 2916 | 24.62 (22.71 ,26.63) |
| 2015-2016 | 2886 | 24.81 (22.48 ,27.31) |
| 2017-2018 | 2828 | 25.89 (24.05 ,27.83) |
| 2019-2020 | 4562 | 24.67 (22.91 ,26.53) |
| Race, % |  |  |
| Mexican American | 1717 | 7.42 (6.02 ,9.12) |
| Non-Hispanic White | 4870 | 65.77 (62.42 ,68.96) |
| Non-Hispanic Black | 3140 | 11.61 (9.96 ,13.48) |
| Other Hispanic | 1404 | 6.17 (5.31 ,7.16) |
| Other Race | 2061 | 9.03 (7.99 ,10.20) |
| Educational level, % |  |  |
| <High school | 2921 | 13.53 (12.22 ,14.96) |
| High school | 3047 | 24.19 (22.76 ,25.68) |
| Some college or more | 7224 | 62.28 (60.23 ,64.29) |
| Family income, % |  |  |
| Low | 4138 | 20.32 (18.57 ,22.19) |
| Medium | 5059 | 34.67 (33.13 ,36.25) |
| High | 3995 | 45.01 (42.49 ,47.55) |
| Heavy drinker, % |  |  |
| No | 8833 | 70.75 (69.31 ,72.15) |
| Yes | 1859 | 14.45 (13.41 ,15.56) |
| Missing | 2500 | 14.80 (13.77 ,15.89) |
| Smoker, % |  |  |
| No | 10464 | 80.12 (78.90 ,81.28) |
| Yes | 2728 | 19.88 (18.72 ,21.10) |
| BMI, kg/m2 | 13192 | 30.58 (30.34 ,30.81) |
| Uric acid, umol/l | 13192 | 326.56 (324.43 ,328.69) |
| HDL-C, mmol/l | 13192 | 1.38 (1.37 ,1.40) |
| TC, mmol/l | 13192 | 5.04 (5.01 ,5.08) |
| TG, mmol/l | 13192 | 1.79 (1.76 ,1.83) |
| BUN, mmol/l | 13192 | 5.27 (5.21 ,5.34) |
| ALT, u/l | 13192 | 25.31 (24.86 ,25.77) |
| AST, u/l | 13192 | 24.39 (24.04 ,24.75) |
| glycohemoglobin, % | 13192 | 5.77 (5.74 ,5.80) |
| eGFR, mL/min per  1.73m^2^ | 13192 | 90.30 (89.59 ,91.00) |
| Cancer, % | 11680 | 86.78 (85.83 ,87.68) |
| No | 1512 | 13.22 (12.32 ,14.17) |
| Yes |  |  |
| ASCVD, % | 11717 | 90.34 (89.53 ,91.09) |
| No | 1475 | 9.66 (8.91 ,10.47) |
| Yes |  |  |
| DM, % | 10410 | 83.66 (82.67 ,84.61) |
| No | 2782 | 16.34 (15.39 ,17.33) |
| Yes |  |  |
| Osteoporosis, % | 5511 | 39.63 (37.42 ,41.88) |
| No | 446 | 3.34 (2.87 ,3.88) |
| Yes | 7235 | 57.03 (54.56 ,59.48) |
| Missing |  |  |
| Stroke, % | 12576 | 96.47 (96.12 ,96.78) |
| No | 616 | 3.53 (3.22 ,3.88) |
| Yes |  |  |
| Heart attack, % | 12559 | 95.99 (95.42 ,96.49) |
| No | 633 | 4.01 (3.51 ,4.58) |
| Yes |  |  |
| CHF, % | 12737 | 97.50 (97.16 ,97.80) |
| No | 455 | 2.50 (2.20 ,2.84) |
| Yes |  |  |
| Walk or bicycle, % | 10322 | 81.01 (79.67 ,82.29) |
| No | 2870 | 18.99 (17.71 ,20.33) |
| Yes |  |  |
| Work activity, % | 7447 | 51.18 (49.81 ,52.55) |
| No | 2756 | 24.56 (23.23 ,25.95) |
| Moderate | 2395 | 19.93 (18.75 ,21.16) |
| Both | 594 | 4.33 (3.74 ,5.00) |
| Vigorous |  |  |
| Recreational activity, % | 7284 | 49.04 (46.99 ,51.09) |
| No | 3403 | 29.24 (27.61 ,30.93) |
| Moderate | 1765 | 15.62 (14.30 ,17.04) |
| Both | 740 | 6.10 (5.44 ,6.82) |
| Vigorous |  |  |
| Antiplatelet agents, % | 12754 | 97.24 (96.79 ,97.62) |
| No | 438 | 2.76 (2.38 ,3.21) |
| Yes |  |  |
| Statins, % | 10140 | 78.44 (77.15 ,79.68) |
| No | 3052 | 21.56 (20.32 ,22.85) |
| Yes |  |  |
| Antihypertensive agents, % | 12415 | 94.62 (94.13 ,95.07) |
| No | 777 | 5.38 (4.93 ,5.87) |
| Yes | 11680 | 86.78 (85.83 ,87.68) |
| Hypertension, % |  |  |
| No | 6596 | 51.34 (49.80 ,52.88) |
| Yes | 6596 | 48.66 (47.12 ,50.20) |
| OA |  |  |
| No | 12973 | 97.92 (97.51 ,98.27) |
| Yes | 219 | 2.08 (1.73 ,2.49) |

Variables are presented as weighted percentage (%) or mean (95%CI).

BMI, body mass index; HDL-C, high-density lipoprotein cholesterol; TC, total cholesterol; TG, triglyceride; BUN, blood urea nitrogen; ALT, alanine aminotransferase; AST, aspartate transaminase; eGFR, estimated glomerular filtration rate; ASCVD, arteriosclerotic cardiovascular disease;DM, diabetes mellitus; CHF, congestive heart failure; OA, osteoarthritis; CI, confidence interval.

**Table S4.** The Association of OA and Hypertension in NHANES 2013-2020 after Propensity Score Matching.

| **Model** | **OR (95% CI)** | ***P* value** |
| --- | --- | --- |
| Model 1 | 1.30 (0.94, 1.81) | 0.112 |
| Model 2 | 1.12 (0.78, 1.61) | 0.534 |

OR, odds ratio; CI, confidence interval.

Model 1: adjusted for none.

Model 2: adjusted for age, gender, body mass index, high-density lipoprotein cholesterol, triglyceride, blood urea nitrogen, alanine aminotransferase, aspartate transaminase, glycohemoglobin, estimated glomerular filtration rate, family income, uric acid, arteriosclerotic cardiovascular disease, diabetes mellitus, stroke, heart attack,congestive heart failure, antiplatelet agents, recreational activity and statins.

**Table S5.** The Horizontal Pleiotropy for Genetic Instruments.

| **Exposure** | **Outcome** | **Egger Intercept** | **Se** | ***P* value** |
| --- | --- | --- | --- | --- |
| Knee OA | Hypertension | 0.016 | 0.013 | 0.217 |
| Hip OA | Hypertension | 0.009 | 0.007 | 0.218 |
| Knee or hip OA | Hypertension | -0.002 | 0.012 | 0.874 |
| OA from UKB | Hypertension | 0.019 | 0.023 | 0.451 |
| OA from UKB (male) | Hypertension | 0.006 | 0.012 | 0.628 |
| OA from UKB (female) | Hypertension | 0.000 | 0.014 | 0.998 |
| Hypertension | Knee OA | 0.004 | 0.004 | 0.329 |
| Hypertension | Hip OA | 0.009 | 0.006 | 0.123 |
| Hypertension | Knee or hip OA | 0.007 | 0.004 | 0.077 |
| Hypertension | OA from UKB | 0.000 | 0.000 | 0.092 |
| Hypertension | OA from UKB (male) | 0.000 | 0.000 | 0.142 |
| Hypertension | OA from UKB (female) | 0.001 | 0.000 | 0.144 |

OA, osteoarthritis; UKB, UK Biobank; se, standard error.

**Table S6.** The Heterogeneity for Genetic Instruments.

| **Exposure** | **Outcome** | **Method** | **Q** | ***P* value** |
| --- | --- | --- | --- | --- |
| Knee OA | Hypertension | MR Egger | 79.99 | <0.001 |
| Knee OA | Hypertension | IVW | 86.49 | <0.001 |
| Hip OA | Hypertension | MR Egger | 67.77 | <0.001 |
| Hip OA | Hypertension | IVW | 72.10 | <0.001 |
| Knee or hip OA | Hypertension | MR Egger | 88.72 | <0.001 |
| Knee or hip OA | Hypertension | IVW | 88.81 | <0.001 |
| OA from UKB | Hypertension | MR Egger | 7.09 | 0.313 |
| OA from UKB | Hypertension | IVW | 7.85 | 0.346 |
| OA from UKB (male) | Hypertension | MR Egger | 8.38 | 0.079 |
| OA from UKB (male) | Hypertension | IVW | 8.96 | 0.111 |
| OA from UKB (female) | Hypertension | MR Egger | 2.22 | 0.330 |
| OA from UKB (female) | Hypertension | IVW | 2.22 | 0.529 |
| Hypertension | Knee OA | MR Egger | 235.89 | <0.001 |
| Hypertension | Knee OA | IVW | 237.66 | <0.001 |
| Hypertension | Hip OA | MR Egger | 247.58 | <0.001 |
| Hypertension | Hip OA | IVW | 252.23 | <0.001 |
| Hypertension | Knee or hip OA | MR Egger | 268.31 | <0.001 |
| Hypertension | Knee or hip OA | IVW | 274.95 | <0.001 |
| Hypertension | OA from UKB | MR Egger | 142.53 | 0.110 |
| Hypertension | OA from UKB | IVW | 145.88 | 0.087 |
| Hypertension | OA from UKB (male) | MR Egger | 150.41 | 0.077 |
| Hypertension | OA from UKB (male) | IVW | 153.00 | 0.065 |
| Hypertension | OA from UKB (female) | MR Egger | 154.13 | 0.051 |
| Hypertension | OA from UKB (female) | IVW | 156.75 | 0.043 |

OA, osteoarthritis; UKB, UK Biobank; IVW, inverse variance weighted.

**Table S7.** The Causal Effect of OA on Hypertension.

| **Exposure** | **Method** | **OR** | **Low_95%CI** | **Up_95%CI** | ***P* value** |
| --- | --- | --- | --- | --- | --- |
| Knee OA | MR Egger | 0.787 | 0.520 | 1.192 | 0.271 |
| Knee OA | Weighted median | 0.975 | 0.902 | 1.055 | 0.531 |
| Knee OA | IVW | 1.024 | 0.931 | 1.126 | 0.626 |
| Knee OA | Simple mode | 0.953 | 0.819 | 1.110 | 0.545 |
| Knee OA | Weighted mode | 0.948 | 0.848 | 1.060 | 0.360 |
| Hip OA | MR Egger | 0.896 | 0.762 | 1.054 | 0.199 |
| Hip OA | Weighted median | 0.983 | 0.935 | 1.033 | 0.494 |
| Hip OA | IVW | 0.990 | 0.941 | 1.042 | 0.704 |
| Hip OA | Simple mode | 0.962 | 0.880 | 1.051 | 0.400 |
| Hip OA | Weighted mode | 0.973 | 0.911 | 1.040 | 0.433 |
| Knee or hip OA | MR Egger | 1.041 | 0.675 | 1.605 | 0.859 |
| Knee or hip OA | Weighted median | 1.011 | 0.924 | 1.107 | 0.804 |
| Knee or hip OA | IVW | 1.005 | 0.915 | 1.105 | 0.911 |
| Knee or hip OA | Simple mode | 1.086 | 0.871 | 1.354 | 0.472 |
| Knee or hip OA | Weighted mode | 1.057 | 0.882 | 1.267 | 0.552 |
| OA from UKB | MR Egger | 0.006 | 0.000 | 925.707 | 0.433 |
| OA from UKB | Weighted median | 0.746 | 0.148 | 3.761 | 0.723 |
| OA from UKB | IVW | 0.796 | 0.233 | 2.714 | 0.715 |
| OA from UKB | Simple mode | 0.984 | 0.060 | 16.036 | 0.991 |
| OA from UKB | Weighted mode | 0.899 | 0.085 | 9.471 | 0.932 |
| OA from UKB (male) | MR Egger | 0.843 | 0.032 | 22.559 | 0.924 |
| OA from UKB (male) | Weighted median | 2.871 | 0.790 | 10.435 | 0.109 |
| OA from UKB (male) | IVW | 1.874 | 0.533 | 6.596 | 0.328 |
| OA from UKB (male) | Simple mode | 6.367 | 0.623 | 65.021 | 0.179 |
| OA from UKB (male) | Weighted mode | 6.367 | 0.905 | 44.810 | 0.122 |
| OA from UKB (female) | MR Egger | 0.793 | 0.016 | 39.081 | 0.918 |
| OA from UKB (female) | Weighted median | 1.051 | 0.249 | 4.433 | 0.946 |
| OA from UKB (female) | IVW | 0.789 | 0.236 | 2.636 | 0.701 |
| OA from UKB (female) | Simple mode | 1.187 | 0.179 | 7.866 | 0.870 |
| OA from UKB (female) | Weighted mode | 1.094 | 0.179 | 6.697 | 0.929 |

OA, osteoarthritis; UKB, UK Biobank; IVW, inverse variance weighted; OR, odds ratio; CI, confidence interval.

**Table S8.** The Causal Effect of Hypertension on OA.

| **Outcome** | **Method** | **OR** | **Low_95%CI** | **Up_95%CI** | ***P* value** |
| --- | --- | --- | --- | --- | --- |
| Knee OA | MR Egger | 0.925 | 0.786 | 1.089 | 0.350 |
| Knee OA | Weighted median | 0.995 | 0.936 | 1.059 | 0.886 |
| Knee OA | IVW | 0.999 | 0.949 | 1.052 | 0.981 |
| Knee OA | Simple mode | 1.013 | 0.873 | 1.175 | 0.866 |
| Knee OA | Weighted mode | 1.007 | 0.879 | 1.154 | 0.920 |
| Hip OA | MR Egger | 0.838 | 0.679 | 1.032 | 0.099 |
| Hip OA | Weighted median | 1.035 | 0.959 | 1.117 | 0.377 |
| Hip OA | IVW | 0.980 | 0.917 | 1.047 | 0.548 |
| Hip OA | Simple mode | 1.063 | 0.875 | 1.290 | 0.541 |
| Hip OA | Weighted mode | 1.056 | 0.892 | 1.251 | 0.528 |
| Knee or hip OA | MR Egger | 0.875 | 0.759 | 1.009 | 0.068 |
| Knee or hip OA | Weighted median | 1.013 | 0.961 | 1.068 | 0.632 |
| Knee or hip OA | IVW | 0.989 | 0.946 | 1.035 | 0.639 |
| Knee or hip OA | Simple mode | 1.049 | 0.907 | 1.213 | 0.522 |
| Knee or hip OA | Weighted mode | 1.021 | 0.909 | 1.146 | 0.729 |
| OA from UKB | MR Egger | 0.991 | 0.982 | 1.000 | 0.050 |
| OA from UKB | Weighted median | 0.997 | 0.993 | 1.002 | 0.220 |
| OA from UKB | IVW | 0.998 | 0.995 | 1.001 | 0.251 |
| OA from UKB | Simple mode | 1.000 | 0.989 | 1.011 | 0.980 |
| OA from UKB | Weighted mode | 0.997 | 0.987 | 1.006 | 0.501 |
| OA from UKB (male) | MR Egger | 0.992 | 0.980 | 1.004 | 0.182 |
| OA from UKB (male) | Weighted median | 1.000 | 0.995 | 1.005 | 0.949 |
| OA from UKB (male) | IVW | 1.000 | 0.997 | 1.004 | 0.847 |
| OA from UKB (male) | Simple mode | 1.003 | 0.990 | 1.017 | 0.638 |
| OA from UKB (male) | Weighted mode | 0.994 | 0.985 | 1.004 | 0.255 |
| OA from UKB (female) | MR Egger | 0.989 | 0.975 | 1.002 | 0.103 |
| OA from UKB (female) | Weighted median | 0.999 | 0.993 | 1.005 | 0.716 |
| OA from UKB (female) | IVW | 0.998 | 0.994 | 1.003 | 0.431 |
| OA from UKB (female) | Simple mode | 0.996 | 0.980 | 1.012 | 0.594 |
| OA from UKB (female) | Weighted mode | 0.998 | 0.983 | 1.012 | 0.740 |

OA, osteoarthritis; UKB, UK Biobank; IVW, inverse variance weighted; OR, odds ratio; CI, confidence interval.
